# Successful β-blocker introduction under intra-aortic balloon pumping and ivabradine in a patient with new-onset dilated cardiomyopathy and pulsus alternans: a case report

**DOI:** 10.1093/ehjcr/ytad620

**Published:** 2023-12-12

**Authors:** Takeshi Kashimura, Mitsuo Ishizuka, Komei Tanaka, Takayuki Inomata

**Affiliations:** Department of Cardiovascular Medicine, Niigata University Medical and Dental Hospital, 1-754 Asahimachi-dori, Chuo-ku, Niigata-city 951-8520, Japan; Department of Advanced Cardiopulmonary Vascular Therapeutics, Niigata University Graduate School of Medical and Dental Sciences, 1-757 Asahimachi-dori, Chuo-ku, Niigata-city 951-8510, Japan; Department of Cardiovascular Medicine, Niigata University Medical and Dental Hospital, 1-754 Asahimachi-dori, Chuo-ku, Niigata-city 951-8520, Japan; Department of Cardiology, Niigata City General Hospital, Niigata-city, Japan; Department of Cardiovascular Medicine, Niigata University Medical and Dental Hospital, 1-754 Asahimachi-dori, Chuo-ku, Niigata-city 951-8520, Japan

**Keywords:** Case report, Pulsus alternans, Mechanical alternans, Left ventricular reverse remodelling, Ivabradine, Beta-blocker, Intra-aortic balloon pump, Calcium overload

## Abstract

**Background:**

Pulsus alternans has been considered a sign of poor prognosis in patients undergoing treatments for heart failure. However, it may be overlooked in patients with intra-aortic balloon pumps (IABPs). The use of IABP and ivabradine for a β-blocker introduction in a patient with dilated cardiomyopathy (DCM) and pulsus alternans and its consequence have never been reported.

**Case summary:**

In a 16-year-old high school boy with idiopathic DCM [left ventricular end-diastolic diameter (LVDd), 72 mm; left ventricular ejection fraction (LVEF), 18%], the introduction of carvedilol therapy failed, causing cardiogenic shock under inotropes. Therefore, an IABP support was provided, and he was transferred to our hospital. The arterial pressure waveform under IABP demonstrated pulsus alternans with sinus tachycardia at 135/min. Ivabradine reduced the heart rate to ∼100/min and eliminated the pulsus alternans neither decreasing the cardiac index nor increasing the pulmonary artery wedge pressure. Subsequently, carvedilol was reintroduced, and IABP and inotropes were discontinued. Then, 112 days after his transfer to our hospital, left ventricular reverse remodelling was confirmed (LVDd, 54 mm; LVEF, 44%), and he returned to school. The carvedilol dose reached 20 mg/day in 4 months after discharge, and further improvement was observed a year after discharge (LVDd, 54 mm; LVEF, 52%).

**Discussion:**

Pulsus alternans is considered a predictor of poor prognosis. However, IABP and ivabradine may stabilize the haemodynamics in pulsus alternans, leading to a successful β-blocker introduction.

**Learning points**

- Pulsus alternans, a predictor of poor prognosis of heart failure, may be overlooked during intra-aortic balloon pumping.
- In patients with new-onset dilated cardiomyopathy with sinus tachycardia and pulsus alternans, intra-aortic balloon pump and ivabradine may stabilize haemodynamics, leading to a successful β-blocker introduction.

## Introduction

Pulsus alternans, or mechanical alternans, is the alternating arterial pressure with a regular heart rhythm, often detected in patients with decompensated heart failure.^1,2^ Pulsus alternans is considered a sign of low cardiac output or compromised perfusion^3^ and a poor prognosis.^1,4^ However, patients with dilated cardiomyopathy (DCM) and pulsus alternans have been reported to achieve left ventricular reverse remodelling after starting β-blockers.^5^ In this article, we present the case of a patient with DCM and pulsus alternans, who was initially intolerant to a β-blocker. β-Blocker therapy was initiated, supported by an intra-aortic balloon pump (IABP) and ivabradine, and left ventricular reverse remodelling was subsequently achieved.

## Summary figure

**Table:** 

−2 months	Dyspnoea on exertion. LVDd = 72 mm, LVEF = 18%.
	Prescribed enalapril, spironolactone, and azosemide
−1 month	Admitted to the previous hospital.
−3 days	Carvedilol 1.25 mg/day caused cardiogenic shock despite the administration of dobutamine and milrinone.
	Carvedilol was abandoned, and IABP was introduced.
Day 0	Transferred to our hospital. LVDd = 72 mm, LVEF = 17%.
	Sinus tachycardia at 135/min and pulsus alternans
	Ivabradine was introduced.
Day 2	The heart rate decreased to ∼100/min.
	Pulsus alternans disappeared.
Day 11–14	Carvedilol was reintroduced and up-titrated to 1.25 mg/day.
Day 19	Intra-aortic balloon pump was withdrawn.
Day 69	The carvedilol dose reached 5 mg/day.
Day 91–103	Dobutamine and milrinone were withdrawn.
Day 112–120	LVDd = 53 mm, LVEF = 44%.
	Discharged and returned to school.
+4 months	The carvedilol dose reached 20 mg/day.
+1 year	LVDd = 54 mm, LVEF = 52%.

## Case presentation

A 16-year-old Japanese high school student with no medical history or prior medication, whose father had been diagnosed with idiopathic DCM, noticed dyspnoea on exertion 2 months before being transferred to our hospital. He went to a hospital and the echocardiogram revealed an enlarged and diffusely hypokinetic left ventricle, with a left ventricular end-diastolic diameter (LVDd) of 72 mm, end-diastolic volume (LVEDV) of 269 mL, and left ventricular ejection fraction (LVEF) of 18%. Enalapril, spironolactone, and azosemide did not improve his symptoms, so he was admitted to the hospital a month before transfer. His heart rate (HR) and blood pressure (BP) were 118/min and 96/55 mmHg, respectively. A right heart catheterization was performed, which revealed mean pulmonary artery wedge pressure (mPAWP) of 33 mmHg, pulmonary artery pressure (PAP) of 51/32(40) mmHg, mean right atrial pressure (mRAP) of 20 mmHg, the pulmonary artery O_2_ saturation (SvO_2_) of 41.3%, and cardiac index (CI) of 1.44 L/min/m^2^. Coronary angiography showed no coronary artery disease, and endomyocardial biopsy showed mild interstitial fibrosis without leucocyte infiltration. Cardiac magnetic resonance imaging, including gadolinium enhancement, revealed no abnormal signals in the myocardium. Blood test results, including troponin T, thyroid function, and vitamin B1, did not suggest secondary sinus tachycardia or cardiomyopathy, and he was diagnosed with idiopathic DCM.

During a month’s hospital stay, carvedilol was introduced at 0.625 mg once daily and titration to 1.25 mg once daily under intravenous administration of dobutamine at 1 μg/kg/min and milrinone at 0.125 μg/kg/min caused lassitude and nausea. The carvedilol dose was maintained at 0.625 mg daily. The second attempt to increase the carvedilol dose to 1.25 mg once daily with intravenous dobutamine at 2 μg/kg/min and milrinone at 0.25 μg/kg/min again caused lassitude and nausea. Carvedilol was abandoned. Right heart catheterization under intravenous administration of dobutamine and milrinone showed haemodynamics consistent with cardiogenic shock: HR, 125/min; arterial pressure (AP), 97/52(67) mmHg; mPAWP, 33 mmHg; PAP, 42/30(35) mmHg; mRAP, 19 mmHg; SvO_2_, 35.4%; CI, 1.40 L/min/m^2^; lactate 2.61 mmol/L (normal range; 0.44–1.78 mmol/L). An IABP was introduced, and haemodynamics improved: HR, 114/min; AP, 88/64(76) mmHg; PAP, 30/20(24) mmHg; mPAWP, not measured; mRAP, 13 mmHg; SvO_2_, 59.0%; and CI, 1.70 L/min/m^2^; lactate 1.46 mmol/L. Three days later, he was transferred to our hospital.

**Figure 1:**
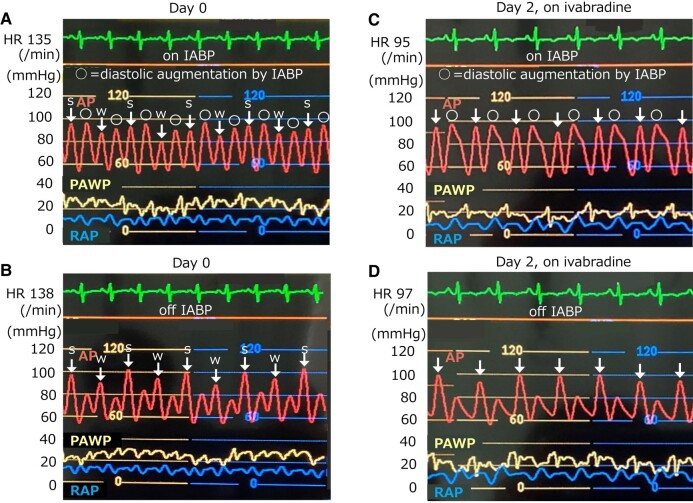
Haemodynamic parameters before and after ivabradine. On the transfer day, the heart rate was 135/min, and arterial pressure waves of the left ventricular ejection showed pulsus alternans (*A*, white arrows; s, strong beat; W, weak beat). The subsequent diastolic augmentation waves by IABP also showed pulsus alternans (*A*, white circles). Brief cessation of IABP made pulsus alternans clearer (*B*, white arrows), despite the prominent dicrotic notches and reflection waves. Note that the amplitude of the pulsus alternans, or the difference between strong and weak beats, stayed at ∼10 mmHg, regardless of on IABP or off IABP. Ivabradine 2.5 mg twice daily reduced his heart rate to 95/min within 2 days. The arterial waves showed that pulsus alternans disappeared under IABP support (*C*, white arrows) and was not caused by a brief cessation of IABP (*D*, white arrows). Respiratory fluctuations of AP were parallel to PAWP. AP, arterial pressure; PAWP, pulmonary artery wedge pressure; RAP, right atrial pressure.

**Figure 2:**
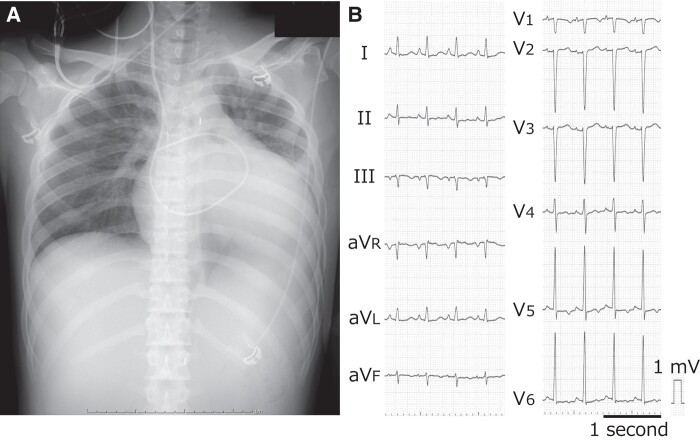
Chest radiograph and electrocardiogram at the transfer to our hospital.

*Figure 1A* shows the haemodynamics after transfer: HR, 135/min; AP, 92/56(74) mmHg under IABP; mPAWP, 24 mmHg; PAP, 40/25(31) mmHg; mRAP, 10 mmHg; SvO_2_, 55.4%; and CI, 2.18 L/min/m^2^. Arterial pressure waves indicated pulsus alternans (*Figure 1A* and *B*, white arrows). Precordial pulsations and a shift of the apex to the left were apparent. No heart murmur or peripheral oedema was detected. Chest radiography and electrocardiogram detected marked cardiomegaly, sinus tachycardia, and mild ST-T abnormalities without T-wave alternans (*Figure 2A* and *B*). Echocardiographic findings did not improve, with the exception of valve regurgitation [LVDd, 76 mm; LVEDV, 304 mL; LVEF, 17%; mild-to-moderate mitral regurgitation (MR); and mild-to-moderate tricuspid regurgitation (TR)].

**Figure 3:**
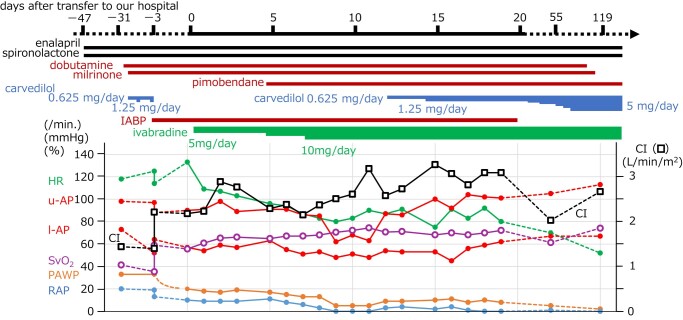
Clinical course. CI, cardiac index; HR, heart rate; l-AP, lower arterial pressure; PAWP, pulmonary artery wedge pressure; RAP, right atrial pressure; SvO_2_, O_2_ saturation of mixed vein; U-AP, upper arterial pressure.

**Figure 4:**
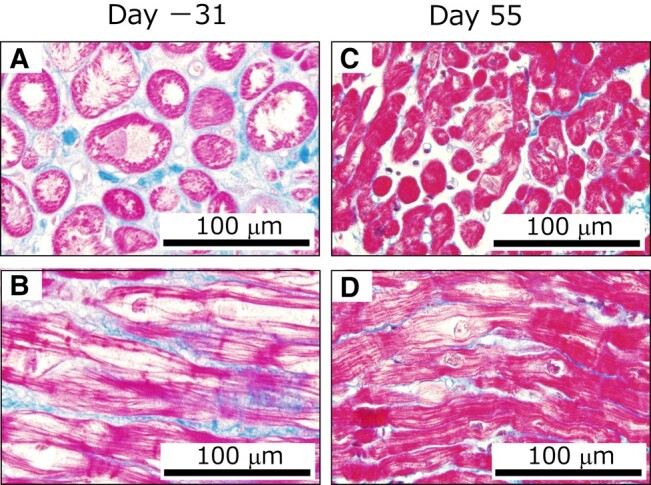
Endomyocardial biopsy examination. Myocardial hypertrophy and vacuolization of left ventricular tissue 31 days after the transfer (*A*, *B*) and milder myocardial hypertrophy and vacuolization of right ventricular tissue on Day 55 (*C*, *D*) were observed with Masson’s trichrome staining.

We considered his excessive tachycardia should be controlled; however, the negative inotropic effect of a β-blocker should be avoided. Ivabradine was introduced at a dose of 2.5 mg twice a day. Two days later, his HR decreased to ∼100/min, without decreasing SvO_2_ or CI, and PAWP decreased without change in diuretics (*Figure 3*), as follows: HR, 107/min; AP, 98/59(78) mmHg; mPAWP, 17 mmHg; PAP, 40/22(29) mmHg; mRAP, 9 mmHg; SvO_2_, 65.3％; and CI, 2.84 L/min/m^2^. Furthermore, arterial waveforms revealed the disappearance of pulsus alternans (*Figure 1C* and *D*). On Day 4, pimobendan was added, with the expectation of additional haemodynamic improvement through a calcium-sensitising effect, and the dose of ivabradine was increased. Carvedilol was reintroduced on Day 12 and increased to 1.25 mg/day on Day 14. The IABP was removed on Day 19. However, he did not complain of dyspnoea. For approximately one month, he experienced orthostatic hypotension and nocturnal hypotension with polyuria despite discontinuation of diuretics, requiring intravenous infusion. Therefore, enalapril was not replaced with angiotensin receptor-neprilysin inhibitor, and sodium-glucose cotransporter-2 inhibitor was not added. On Day 55, the right heart catheterization data were as follows: HR, 73/min; AP, 105/67(79) mmHg; mPAWP, 5 mmHg; PAP, 16/5(9) mmHg; mRAP, 1 mmHg; SvO_2_, 61.4％; and CI, 2.03 L/min/m^2^. Endomyocardial biopsy of the right ventricle on Day 55 showed milder myocardial enlargement and vacuolation (*Figure 4C* and *D*) compared to the biopsy done on the left ventricle at the previous hospital 31 days before transfer (*Figure 4A* and *B*). The dose of carvedilol was titrated gradually and reached 5 mg/day on Day 69. Then, dobutamine and milrinone were withdrawn on Days 91 and 103, respectively. On Day 112, echocardiography revealed left ventricular reverse remodelling (LVDd, 53 mm; LVEDV, 132 mL; LVEF, 44%; mild MR; and mild TR) (see Supplementary material online, *Video S1*). His haemodynamics improved further on Day 119: HR, 55/min; BP, 113/67 mmHg; mPAWP, 2 mmHg; PAP, 14/3(8) mmHg; mRAP, 0 mmHg; SvO_2_, 74.2％; and CI, 2.67 L/min/m^2^. Therefore, he was discharged from our hospital, returned to school, and was prescribed carvedilol 5 mg/day, ivabradine 10 mg/day, enalapril maleate 2.5 mg/day, spironolactone 25 mg/day, and pimobendan 5 mg/day. The dose of carvedilol reached 20 mg/day four months later. One year after discharge, LVDd, LVEDV, and LVEF were 54 mm, 138 mL, and 54%, respectively, MR was not detected, and TR was mild.

## Discussion

This case with DCM and pulsus alternans was initially intolerant to the β-blocker; however, an IABP and ivabradine supported initiation of β-blocker therapy, and left ventricular reverse remodelling was subsequently achieved with an angiotensin-converting enzyme inhibitor and a mineral corticoid receptor antagonist.

Kodama *et al*.^5^ reported that DCM patients with pulsus alternans achieved left ventricular reverse remodelling if a β-blocker was successfully introduced. Therefore, pulsus alternans before the introduction of the β-blocker may not always be a sign of a bad prognosis. In this case, IABP was effective in reducing ventricular filling pressure and increasing CI, but did not reduce HR, and pulsus alternans remained.

Tachycardia, one of the mechanisms for compensating left ventricular systolic dysfunction and low cardiac output, is characterized by the frequency and strength of the contraction, known as the force–frequency relationship. The cellular mechanism of this relationship assumes that the more frequently the myocardium depolarizes, the more calcium the myocardium receives, leading to greater muscle contraction.^5–8^ The force–frequency relationship is impaired in patients with DCM,^9^ particularly in those with pulsus alternans.^10^ A heart with pulsus alternans cannot keep up with tachycardia and does not contract strongly enough during tachycardia,^11^ which suggests that a heart with pulsus alternans is loaded with more calcium than the heart can use, in other words, ‘calcium overload’.

An acute effect of ivabradine includes increased ventricular filling and stroke volume of each heartbeat in patients with excessive sinus tachycardia.^12^ In this patient with sinus tachycardia and pulsus alternans, ivabradine was prescribed before starting the use of a β-blocker, considering its negative inotropic effect. Ivabradine decreased HR and eliminated pulsus alternans, without decreasing the CI or increasing ventricular filling pressure. The reduction in ‘calcium overload’ may have led to the absence of an increase in filling pressure.^7,8^ Pimobendan was also possibly beneficial due to its inotropic effect as a calcium sensitizer, reducing the need for more calcium loading despite its calcium loading potential.^13^

It was reported that simultaneous use of ivabradine and β-blocker improved systolic function compared with β-blocker alone at 4 months.^14^ The chronic effects of ivabradine include left ventricular reverse remodelling and improvement in the composite outcome of cardiovascular death or hospital admission due to worsening heart failure in patients with a sinus rate > 70/min, even with basal β-blocker therapy.^15^ Therefore, the good clinical evolution of the case presented may not have been obtained without IABP and ivabradine.

## Supplementary Material

ytad620_Supplementary_Data

## Data Availability

The data underlying this article will be shared on reasonable request to the corresponding author.

## Lead author biography

He completed his MD and PhD at Niigata University, Niigata, Japan, and learned calcium handling as a research fellow at the laboratory of Professor David Eisner, the unit of cardiac physiology of the University of Manchester, Manchester, UK. His current research interests include heart failure, cardiomyopathy, and pulmonary hypertension.

## Supplementary material

**Consent:** The authors confirm that consent was obtained from the patient and his mother prior to submission and publication, including for all figures and information contained in this report in line with the Committee on Publication Ethics (COPE) guidelines.
